# Whole-genome sequencing to control antimicrobial resistance

**DOI:** 10.1016/j.tig.2014.07.003

**Published:** 2014-09

**Authors:** Claudio U. Köser, Matthew J. Ellington, Sharon J. Peacock

**Affiliations:** 1 Department of Medicine, University of Cambridge, Cambridge, UK; 2 Clinical Microbiology and Public Health Laboratory, Public Health England, Cambridge, UK; 3 Cambridge University Hospitals National Health Service Foundation Trust, Cambridge, UK; 4 Wellcome Trust Sanger Institute, Wellcome Trust Genome Campus, Hinxton, UK

**Keywords:** whole-genome sequencing, antibiotic resistance, surveillance, diagnostics

## Abstract

Following recent improvements in sequencing technologies, whole-genome sequencing (WGS) is positioned to become an essential tool in the control of antibiotic resistance, a major threat in modern healthcare. WGS has already found numerous applications in this area, ranging from the development of novel antibiotics and diagnostic tests through to antibiotic stewardship of currently available drugs via surveillance and the elucidation of the factors that allow the emergence and persistence of resistance. Numerous proof-of-principle studies have also highlighted the value of WGS as a tool for day-to-day infection control and, for some pathogens, as a primary diagnostic tool to detect antibiotic resistance. However, appropriate data analysis platforms will need to be developed before routine WGS can be introduced on a large scale.

## Highlights

• Owing to improvements in sequencing technologies, microbial whole-genome sequencing (WGS) has emerged as a central tool to control antibiotic resistance.
• WGS has been used to develop novel antibiotics and diagnostic tests.
• WGS has been key to surveillance and the study of the emergence of antibiotic resistance.
• Rapid WGS has the potential to be used as a tool for infection control in the clinic and, in some cases, as a primary diagnostic tool to detect resistance.

## A modern tool for a growing challenge

The spread of antibiotic resistance constitutes one of the most serious threats to human health [1]. If left unchecked, even ‘minor surgery and routine operations could become high risk procedures’ [2]. As a result, the UK Chief Medical Officer has called for antibiotic resistance to be included in the National Risk Register [2]. The causes of the spread of antibiotic resistance are complex, as are the strategies to combat this threat 3, 4, 5. These include developing novel agents as well as maximising the utility of currently available antibiotics by preventing infections and improving diagnostics. As a result of recent technological advances, microbial WGS has emerged as a tool that could underpin the success of such goals.

A detailed discussion of the various sequencing technologies and their advantages and disadvantages for pathogen WGS can be found elsewhere 6, 7, 8. For the purposes of this review it is merely necessary to appreciate that two classes of sequencers exist. The first can be compared to oil tankers in that they are relatively slow but have a high throughput and low cost (currently approximately $65 per bacterial genome) 6, 9. By contrast, benchtop sequencers are akin to speedboats. They have a lower throughput and higher cost (approximately $150 per genome in the case of the Illumina MiSeq) but can sequence multiple genomes in less than 24 h 6, 10. In fact, recent advances in sample preparation have enabled WGS directly from single bacterial colonies grown under routine diagnostic conditions without the need for subculture to isolate sufficient DNA for sequencing [11]. Together, these sequencers have allowed researchers to interrogate the evolution of antibiotic resistance at an unprecedented scale. For example, a recent study sequenced the genomes of more than 3000 pneumococcal isolates and another sequenced more than 3600 group A streptococci 12, 13. In addition, benchtop sequencers have become sufficiently fast to become primary diagnostic tools rather than tools for research or retrospective surveillance 9, 14. We review here the insights that WGS has provided with regards to antimicrobial resistance. Owing to the rapid expansion of this field, this endeavour must inevitably be a selective one.

## WGS to develop novel antibiotics

WGS has become an essential tool for drug development by enabling the rapid identification of resistance mechanisms, particularly in the context of tuberculosis (TB), which remains a global public health emergency 15, 16. In 2005 the first published use of 454 pyrosequencing (the first second-generation WGS technology) was to identify the F0 subunit of the ATP synthase as the target of bedaquiline, which subsequently became the first representative of a novel class of anti-TB agents to be approved in 40 years 16, 17. This has enabled researchers to sequence this gene in phylogenetically diverse reference collections to ensure that it is conserved across *Mycobacterium canettii* as well as the various lineages and species that comprise the *Mycobacterium tuberculosis* complex (MTBC), the causative agents of TB [18]. This represents an important step because drug candidates are usually only tested against a small number of isolates during the early phases of drug development. Similarly, only a limited number of MTBC genotypes are sampled in clinical trials, depending on where these are conducted [19]. As a result, intrinsically resistant strains might be missed, as has been the case for PA-824, an anti-TB agent in Phase III trials 19, 20, 21.

The early elucidation of resistance mechanisms using WGS also has implications for the design of clinical trials. If resistance mechanisms are discovered that only result in marginally increased minimal inhibitory concentrations (MICs) compared with the wild type MIC distributions, more frequent dosing or higher doses could be employed in clinical trials to overcome this level of resistance. Moreover, the discovery of cross-resistance between agents using WGS can influence the choice of antibiotics that are included in novel regimens. TB is always treated with multiple antibiotics to minimise the chance of treatment failure as a result of the emergence of resistance during treatment [22]. Regimens that contain agents to which a single mutation confers cross-resistance should be avoided if these mutations arise frequently *in vivo*. WGS has recently highlighted that this may be the case with three Phase II trial regimens that contain bedaquiline and clofazimine because the mutational upregulation of an efflux pump confers cross-resistance to both drugs [23].

In addition to being a tool to design clinical trials, WGS has become an increasingly important tool during clinical trials. Specifically, it is increasingly being used to distinguish exogenous reinfection from relapse of the primary infection, which is crucial in assessing the efficacy of the drug or regimens under investigation 24, 25. Traditional epidemiological tools do not always provide the necessary resolution for this purpose. This is due to the fact that they only interrogate minute parts of the genome {e.g., multilocus sequence typing (MLST) of *Pseudomonas aeruginosa* analyses only 0.18% of the genome [26]}. By contrast, WGS interrogates the complete (or near-complete) genetic repertoire of an organism. Therefore, the resolution of WGS is only limited by the rate of evolution of the pathogen and will become the gold standard for clinical trials of new anti-TB agents and other infectious diseases associated with recurrent disease 27, 28.

## WGS for surveillance

Surveillance is the cornerstone in the control of infectious diseases 3, 29. In this context, the ultimate molecular resolution delivered by WGS has provided unprecedented insight into the history of the emergence and spread of antibiotic resistance [30]. In the case of *Mycobacterium abscessus* this has resulted in a paradigm shift in the understanding of its transmission*.* WGS provided strong evidence that this non-tuberculous mycobacterium, which is difficult to treat owing to its intrinsic resistance to many antibiotics, is transmissible between cystic fibrosis (CF) patients in the hospital setting [31]. Transmission occurred despite strict infection control policies that were designed to prevent the acquisition of other pathogens, such as epidemic *P. aeruginosa* (to be discussed below), that are known to be transmissible between CF patients. Importantly, this included the transmission of macrolide-resistant isolates whereas environmental *M. abscessus* isolates are susceptible to the drug [31]. As a result, the national infection control guidelines have been revised to minimise the possibility of further transmission [32]. Moreover, a much larger UK-wide and international study is currently underway to investigate the extent of transmission elsewhere [33].

WGS has highlighted the importance of the international spread of antibiotic-resistant organisms [34]. For example, the epidemic *P. aeruginosa* Liverpool strain has been shown to transmit between CF clinics in the UK and North America [26]. The development of fluoroquinolone resistance in the EMRSA-15 lineage of methicillin-resistant *Staphylococcus aureus* (MRSA) in the mid-1980s preceded the pandemic spread of this variant [35]. Similarly, the acquisition of fluoroquinolone resistance has been identified as a key genetic change linked to the emergence of the two lineages of epidemic *Clostridium difficile* (027/BI/NAP1) during the early 2000s [36].

Large WGS studies have also started to shed light on the interface between humans and animals. Throughout the 1990s, multidrug-resistant *Salmonella* Typhimurium DT104 was responsible for a global epidemic [37]. Contrary to prior tenets, a comparison of longitudinal human and livestock isolates from Scotland demonstrated that human infections were not primarily due to transmission from local animals. Instead, the human and animal populations of Typhimurium DT104 in Scotland were largely distinct, which suggests that other sources of infection, such as imported food, were responsible for the human disease [37]. By contrast, WGS demonstrated that livestock-associated CC398 MRSA originated from human methicillin-susceptible *S. aureus* and acquired methicillin resistance before again crossing the species barrier to cause disease in humans [38].

## WGS to study the emergence of antibiotic resistance

Unlike the aforementioned studies that reconstructed the evolution of antibiotic resistance using historical isolates, WGS has also been used to study the evolution of resistance in real-time under a variety of conditions [39]. For example, Toprak *et al.* developed a microbial cultivation system capable of maintaining a constant antibiotic pressure to monitor the evolution of resistance to trimethoprim, chloramphenicol, or doxycycline over 20 days in *Escherichia coli* [40], whereas Safi *et al.* relied on antibiotic-containing solid medium to study the evolution of ethambutol resistance in MTBC [41]. The latter study not only demonstrated that ethambutol resistance emerges in a stepwise process but also highlighted a novel resistance mechanism. Specifically, a synonymous mutation in *Rv3792*, which would usually be excluded in analyses aiming to identify the genetic basis of resistance [19], was found to increase ethambutol MICs by increasing the expression of the downstream *embC* gene (probably by creating an internal promoter) [41]. Subsequently, an equivalent mechanism was shown to confer isoniazid and ethionamide cross-resistance in MTBC. In that case, a synonymous mutation in *mabA* creates an alternative promoter for *inhA*, the shared target of isoniazid and ethionamide [42].

In addition to elucidating the mechanisms of resistance, WGS has also played a role in measuring the rate at which resistance emerges. Ford *et al.* found that lineage 2 MTBC isolates (comprising the Beijing genotype, the most frequent MTBC genotype globally) acquire rifampicin resistance at an elevated rate compared with lineage 4 strains (which are widespread in Africa, the Americas and Europe) 43, 44, 45. Modelling of these elevated rates suggests that patients infected with drug-susceptible lineage 2 isolates have a 22-fold increased risk of having developed multi-drug resistance (MDR; i.e., resistance to rifampicin and isoniazid) by the time that they are initially diagnosed, which highlights the importance of early and active case detection [45].

WGS has also clarified some of the factors that have allowed the spread of drug-resistant MTBC. Specifically, WGS has identified mutations in the α and β’ subunits of the RNA polymerase that alleviate the fitness costs of resistance to rifampicin, which constitutes the backbone of the DOTS (directly observed treatment, short-course) regimen for drug-susceptible TB [46]. These compensatory mutations are widespread in MDR MTBC isolates in high-incidence countries and are associated with ongoing transmission 22, 47. As a result, these isolates are unlikely to disappear, which underscores the importance of the development and deployment of rapid point-of-care drug-susceptibility testing (DST) assays to identify MDR strains, as opposed to continuing to rely on empiric treatment, as is done in most parts of the world [22].

## WGS to develop diagnostic tests

The knowledge gained from WGS has been instrumental in assessing and, if necessary, improving diagnostic assays for DST. For example, the discovery of *mecC* (a homologue of the methicillin-resistance gene *mecA* in *S. aureus*) using WGS prompted the redesign of clinical diagnostic PCR assays to avoid false-negative results [48]. By contrast, it was found that the phenotypic Vitek 2 DST assay can be adapted to detecting *mecC* strains simply by adjusting the default rules used to interpret the raw data generated by this system [48].

WGS has also started to play an important role in clarifying the reason for the discrepancies between genotypic and phenotypic DST results for MTBC. For most anti-TB agents the specificity of known resistance mechanisms is higher than their sensitivity. This means that the current generation of genotypic DST assays can usually only be used to rule-in resistance as opposed to rule-out resistance/confirm susceptibility 9, 49. For some agents, such as pyrazinamide, false-positive phenotypic DST results are a known problem, but generally the consensus is that so far unknown resistance mechanisms are responsible for the majority of the unexplained phenotypic resistance to many drugs 22, 50. Indeed, several recent large WGS studies have identified more than 70 genes that might be involved in drug resistance [51]. If confirmed, WGS would be the only option to detect changes in these genes given that the current generation of rapid genotypic DST assays only interrogate short stretches of bacterial DNA (discussed in more detail below) [49]. However, deep WGS studies have highlighted that the development of resistance is more dynamic than previously appreciated [52]. In fact, a recent study found 38% of ofloxacin mono-resistant isolates to be heteroresistant [53]. Importantly, the proportion of ofloxacin resistance that could be explained with classical *gyrA* and *gyrB* mutations increased from 58% to 77% using deep sequencing compared with Sanger sequencing [53]. Consequently, the clinical utility of current molecular assays could be greatly increased if their limit of detection was improved compared with phenotypic DST – which is calibrated to detect resistance at 1% or more of the total population [54]. This also means that novel resistance mechanisms account for a smaller proportion of phenotypic resistance than is currently assumed.

In contrast to the above examples of false-negative results generated by current diagnostic assays, WGS has also been used to identify false-positive results. For instance, the analysis of WGS data from a large collection of phylogenetically diverse MTBC isolates demonstrated that a mutation that is interrogated by a genotypic DST assay for ethambutol resistance is not a marker for drug resistance [55]. Instead, the mutation represents a marker for phylogenetically ancestral MTBC, which means that the DST assay would yield more false-positive than true-positive results in countries where these ancestral isolates are dominant [55]. WGS has highlighted similar flaws in the interpretation of genotypic DST results for streptomycin and pyrazinamide 56, 57. This underlines that the MTBC diversity has to be considered when designing and validating genotypic DST assays [19].

## WGS to direct infection control measures in the clinic

The WGS studies discussed so far relied on slow, high-throughput sequencers to minimise the costs of these large retrospective projects. However, the introduction of rapid benchtop sequencers has enabled the prospective use of WGS, which could be used to halt the spread of antibiotic-resistant bacteria. The first organism for which this was achieved was MRSA [58]. Initially, the investigation by Harris *et al.* began as a retrospective study to confirm that an MRSA outbreak had occurred on a special care baby unit. Nevertheless, during the data analysis stage of this project a further infant was found to be potentially colonised with the outbreak strain, despite having been admitted 64 days after the last MRSA-positive patient had left the unit, which had also undergone deep cleaning. Rapid WGS confirmed that the isolate in question was part of the outbreak, and this led to the hypothesis that a member of staff might have reintroduced the outbreak strain after the last gap in the outbreak. Consistent with this prediction, screening of 154 members of staff identified one MRSA carrier who, by further rapid WGS, was confirmed to be colonised with the outbreak strain. The member of staff was relieved of clinical duties and received decolonisation treatment [58]. No further MRSA isolates linked to the outbreak were observed on the unit, and this suggested that the WGS-led intervention brought the outbreak to a halt.

This and other small proof-of-principle studies are being complemented with much larger hospital-wide longitudinal and community studies of *S. aureus* and *C. difficile* to study the detailed spread of these pathogens 10, 59, 60, 61, 62, 63, 64. On a very practical level, these studies will also start to answer the question about how rapid WGS should be used clinically. Currently, approximately one third of *C. difficile* cases in England and only a small fraction of MRSA carriage and invasive disease are typed [65]. It remains to be seen whether a blanket policy of WGS for all cases (as is the case in the UK for MTBC – discussed below) would be cost-effective, or whether WGS would be used in an *ad hoc* manner (e.g., in response to an increase in cases as in the Harris *et al.* study [58]). In effect, it needs to be determined how many cases could be prevented if WGS data were available in real-time and, conversely, what the value of disproving an outbreak is, in which case infection control efforts can be directed elsewhere 10, 66.

## WGS to detect antibiotic resistance in the clinic

Several recent studies have compared phenotypic DST results with the predictions based on WGS data for a variety of bacterial pathogens 67, 68, 69. Further studies that comprise thousands of strains are also underway. In one of these studies, Gordon *et al.* first compared the routine DST results for 12 commonly used antibiotics of 501 isolates of *S. aureus* with WGS data to develop a panel of resistance determinants (chromosomal resistance mutations as well as acquired resistance genes) [69]. This panel was subsequently used to perform WGS DST for a further 491 isolates, which yielded an overall sensitivity and specificity of 0.97 [95% confidence interval (95% CI), 0.95–0.98] and 0.99 (95% CI, 0.99–1), respectively. The overall rate of very major errors and major errors was 0.5% (95% CI, 0.3–0.7%) and 0.7% (95% CI, 0.5–0.9%), respectively. Importantly, the error rates were within the limits set by the FDA for marketing approval (<1.5% very major discrepancy rate and <3% major discrepancy rate) for most antibiotics individually, with the exception of clindamycin and penicillin [69].

Despite the remarkable concordance between genotype and phenotype observed by Gordon *et al.*, WGS (directly from primary samples or pure culture) will not become a primary diagnostic tool to detect antibiotic resistance in the foreseeable future for *S. aureus*, nor for the vast majority of bacterial pathogens for which drug resistance is considered a major threat [9]. Indeed, unbiased metagenomic WGS, albeit technically possible, remains prohibitively expensive as a result of contamination with DNA from other microorganisms and the host 70, 71. Moreover, WGS is less sensitive than culture or PCR when applied directly to clinical samples (particularly faecal samples that are highly mixed) and it can be difficult to assign to which pathogen a plasmid-borne resistance gene belongs [70]. Consequently, traditional alternatives such as PCR, array technologies, and culture will continue to be the technologies of choice, with the exception of unusual clinical circumstances [72]. Even when starting from a pure culture, phenotypic DST (e.g., by disc diffusion or automated liquid-culture methods) remains faster and cheaper than even the recently developed rapid single-colony WGS protocol [11]. Nevertheless, the aforementioned data and algorithms developed by Gordon *et al.* will be useful for routine surveillance of novel resistance mechanisms and quality control (e.g., if an isolate is sequenced for infection control purposes, the genomic DST result could be routinely compared with the phenotypic results, which will already be available by the time the WGS result is obtained, to identify if the right isolate was sequenced or if the phenotypic DST results were correct).

There are two types of clinical scenarios in which rapid WGS could play a role in the context of diagnosing antibiotic resistance. The first applies when the elucidation of the resistance mechanism is clinically relevant 11, 73. One such instance is in linezolid-resistant *Enterococcus faecium* where distinguishing between mutational and plasmid-mediated linezolid resistance can affect the decision to implement enhanced infection control measures [74]. A more complex example in this category is that where carbapenem resistance is detected during screening (e.g., using growth in the presence of an indicator carbapenem [75]). In this context, WGS could be used to distinguish carbapenemase producers, which demand enhanced infection control procedures (e.g., enhanced screening or cohort staffing), from isolates that are carbapenem-resistant by virtue of other mechanisms 73, 75, 76, 77. However, it is important to appreciate that WGS is unlikely to be cost-effective compared with currently available alternatives such as multiplex PCRs or array technology, if used solely to distinguish resistance mechanisms [4]. By contrast, WGS will be superior if the data are also used to help track onward transmission of bacteria or, in the case of a multi-species carbapenem-resistance outbreak, resistance plasmid transfer between bacteria 11, 73, 78, 79. It will also improve the understanding of the basis of non-congruencies between genotypic and phenotypic data, which can occur for some carbapenemase-positive organisms that are phenotypically sensitive but genotypically positive for a carbapenemase gene [80].

The second clinical scenario in which WGS will become a primary diagnostic tool is when phenotypic testing is prohibitively slow. For this reason, genotypic DST using Sanger sequencing of amplicons has been in clinical use for many years for HIV and is increasingly being replaced with deep sequencing to allow the detection of low-frequency mutations (i.e., heteroresistance), to simplify workflows, and to reduce cost [9]. Current efforts to improve WGS protocols will allow DST for all currently available anti-retroviral drugs in a single assay [81]. Phenotypic DST is also slow and technically demanding for MTBC 9, 49. Molecular techniques have accelerated testing for some antibiotics (most notably the Xpert MTB/RIF, which simultaneously identifies MTBC and rifampicin resistance in 2 h directly from sputum, and which has become the front-line diagnostic test in South Africa 16, 82), but the remaining commercial genotypic DST assays have only played a limited role because they only target the most frequent resistance mutations for a limited number of antibiotics [49]. Owing to the increased resolution that WGS provides for epidemiological typing for MTBC at a comparable cost to current methods, rapid WGS will likely be introduced for every case of TB in England, which is a low-prevalence area for TB with 5200 culture-confirmed cases in 2012, to guide contact tracing 83, 84, 85, 86, 87. The same data could be used for genotypic DST of all known resistance mechanisms simultaneously at no additional cost, thereby reducing the time required to diagnose highly drug-resistant cases from weeks to days, depending on the time to culture positivity 9, 49. In this situation phenotypic DST would not have to be performed when well-understood resistance mutations are found. Nevertheless, even with WGS, phenotypic testing will remain necessary to rule out resistance/confirm susceptibility in the near future given that deep WGS to detect resistance mutations at 1% or more of the total population remains too expensive for routine use, and given that novel resistance mechanisms remain to be discovered, as discussed above.

## Concluding remarks

WGS has become an invaluable tool to combat antibiotic resistance. Its greatest, yet unfulfilled, potential is as a primary tool to direct day-to-day infection control in hospitals and the community, as summarised in several recent reviews 9, 14, 66, 83, 88, 89, 90, 91, 92. This is primarily due to the fact that the current tools available for the automation of WGS analysis lack many of the features required for clinical use 9, 93, 94, 95, 96. Once this obstacle has been overcome, the full benefits of WGS as a true platform technology will be realised by providing superior information compared to current methods, all while simplifying workflows and providing unprecedented economies of scale in diagnostic laboratories 9, 11, 73.

## Disclaimer statement

This publication presents independent research supported by the Health Innovation Challenge Fund (HICF-T5-342 and WT098600), a parallel funding partnership between the UK Department of Health and Wellcome Trust. The views expressed in this publication are those of the authors and not necessarily those of the Department of Health, Public Health England, or the Wellcome Trust. Matthew Ellington was funded by Bruker Daltonics to attend a conference. Sharon Peacock has been a consultant for Pfizer and has received funding for travel and accommodation from Illumina.

## Glossary

Deep sequencing: WGS of a pure sample (e.g., DNA extracted from liquid culture that was inoculated from a single bacterial colony) is usually sequenced to at least a 10- to 15-fold depth (i.e., each position in a genome is sequenced 10–15 times). However, when sequencing samples that might be heteroresistant (see below), or might contain multiple strains of the same species, greater sequencing depth is required to identify minority variants or to separate the strains [49]. For example, deep sequencing, albeit of sections of the genome rather than its entirety, is used routinely in clinical applications for genotypic HIV DST (see below) to detect resistances that occur below 15–20% of the total population, which is the limit of detection for Sanger sequencing [9].
Drug-susceptibility testing (DST): the process of classifying an isolate or a mix of isolates from the same species (e.g., in the case of HIV) as susceptible (high likelihood of clinical response) or resistant (low likelihood of clinical response) to an antibiotic or antiviral. In some cases, an intermediate category (clinical success uncertain) is employed [97]. For most pathogens this is achieved using phenotypic methods, whereas for slow-growing organisms, such as HIV, genotypic DST is the standard of care 9, 98. WGS will not replace phenotypic DST for fast-growing organisms given that it would be too expensive, but will enable rapid genotypic DST for MTBC, for which phenotypic alternatives are slow 9, 49.
DST errors: when comparing two DST methods, a very major error occurs if the assay under evaluation gives a susceptible result compared with a resistant result by the reference method. The misclassification of a susceptible reference result as resistant is referred to as a major error. The classification of a susceptible or resistant reference result as intermediate, or vice versa, is known as a minor error [98].
Heteroresistance: a mixture of susceptible and resistant populations, which may arise as a result of a mixed infection with strains with different susceptibility patterns, or the evolution of a resistant subpopulation from a susceptible parental strain during the infection [99]. The ability of different DST methods to detect resistant isolates at a low percentage of the total population varies (see deep sequencing) [54].
Metagenomics: unbiased sequencing from a primary sample (i.e., without isolating or culturing a specific organism). Depending on the type of sample, it may be highly mixed with several bacterial and viral species as well as host nucleic acid. Provided that the mixed DNA is sequenced to sufficient depth, the genome of an organism can be recovered directly from a clinical sample [70].
Whole-genome sequencing (WGS): modern WGS relies on one or a combination of second- or third-generation sequencing technologies [6]. Although the entire genome is sequenced, it cannot be assembled completely if a genome contains repeated stretches of DNA that are longer than the length of DNA that can be sequenced in a single read by the sequencing technology employed [100]. Additional parts of the genome may be excluded for some analyses. For example, only the core genome that is present in all isolates in question is used for phylogenetic analyses, which represents about 80% of the entire genome for *Clostridium difficile*[62].
